# Supplementary material for: Metabolic dysregulation impairs lymphocyte function during severe SARS-CoV-2 infection
Source: Commun Biol. 2023 Apr 7;6:374. doi: 10.1038/s42003-023-04730-4 (PMC10080180; doi:10.1038/s42003-023-04730-4)
Supplement: Supplementary file 2 — Supplementary Material [file 42003_2023_4730_MOESM2_ESM.pdf]

Supplemental Figure 1. Representative flow cytometry gating strategy. Gating strategy to define population as conducted using Flowjo10.0. $CD8^+T_M$ was defined as the combination of effector memory (EM) and central memory (CM) $CD8^+$Tc populations. $CD8^+T_{EM}$ and $CD8^+T_{CM}$ were defined using either CCR7 and CD62L, or CD45RA and CD62L.

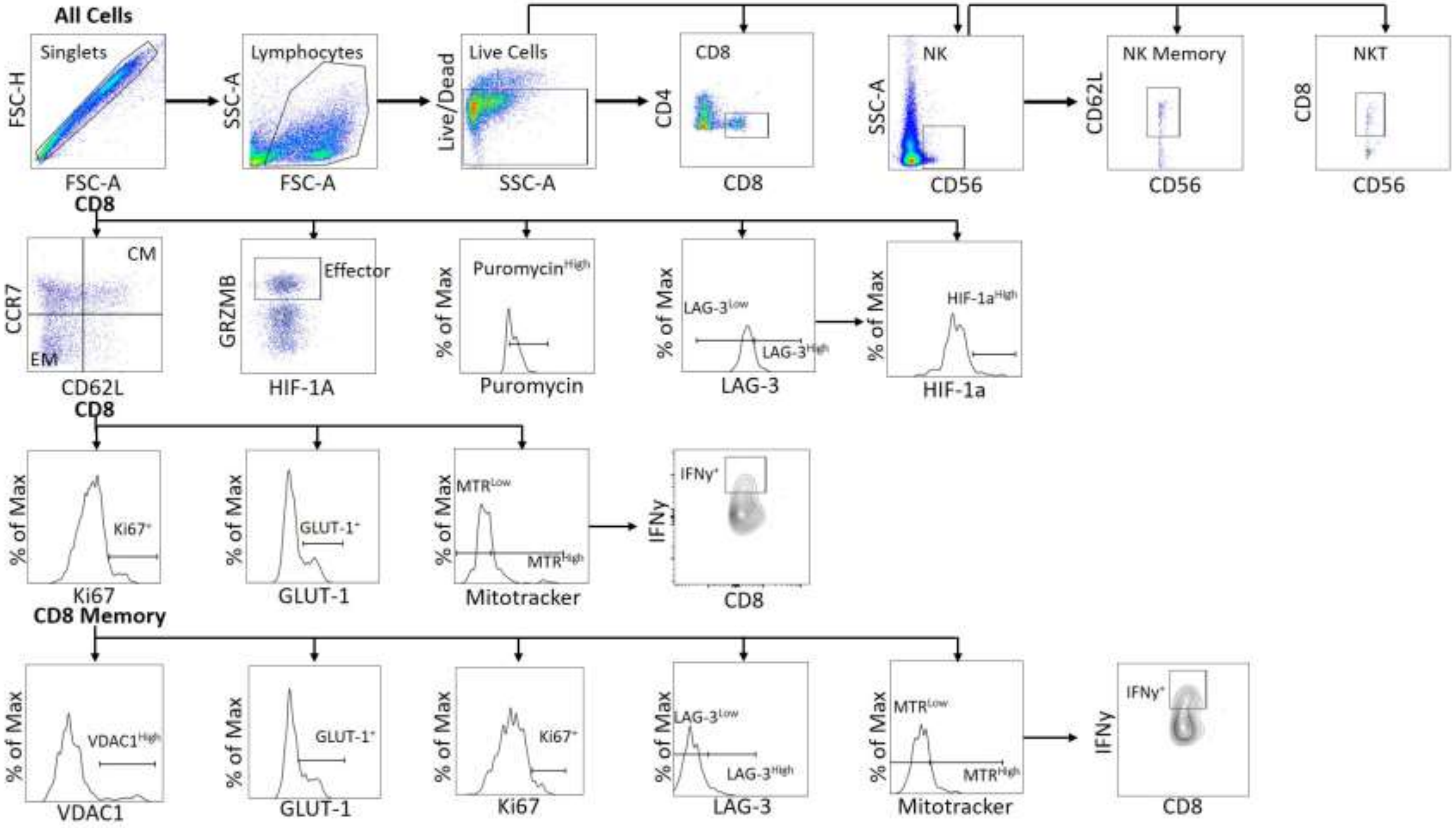

Supplemental Figure 2. Unsupervised clustering for CD8$^+$Tc and NK of patient PBMCS. (**a**) UMAP projection demonstrating unsupervised clusters generated by FlowSOM. (**b**) Heatmap demonstrating canonical marker expression for labelled populations identified using unsupervised clustering. (**c**) UMAP projection of patient PBMCs overlayed with scaled expression of markers for unsupervised clustering.

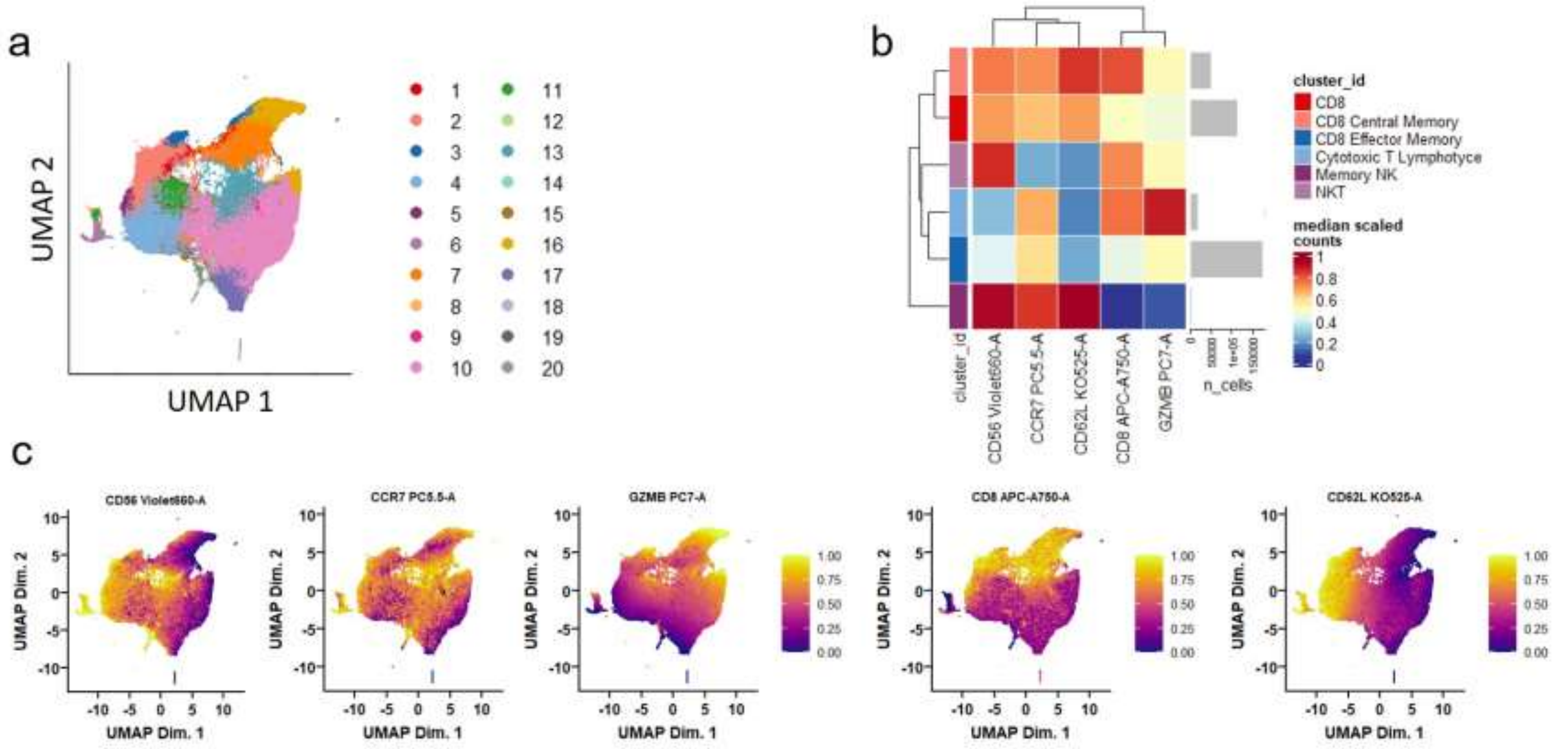

Supplemental Figure 3. Heatmap displaying expression of genes used to annotate unsupervised clusters after sample integration. a. FindAllMarkers function in Seurat was used to identify the top genes specific to each annotated cell population. CD4, macrophage, peripheral monocyte, nonciliated epithelium, B, CD8, mDC. **b.** pDC, proliferating T, plasma, ciliated epithelium, neutrophil, and mast cells. A heatmap displaying the average gene expression was generated using the DoHeatmap function in Seurat.

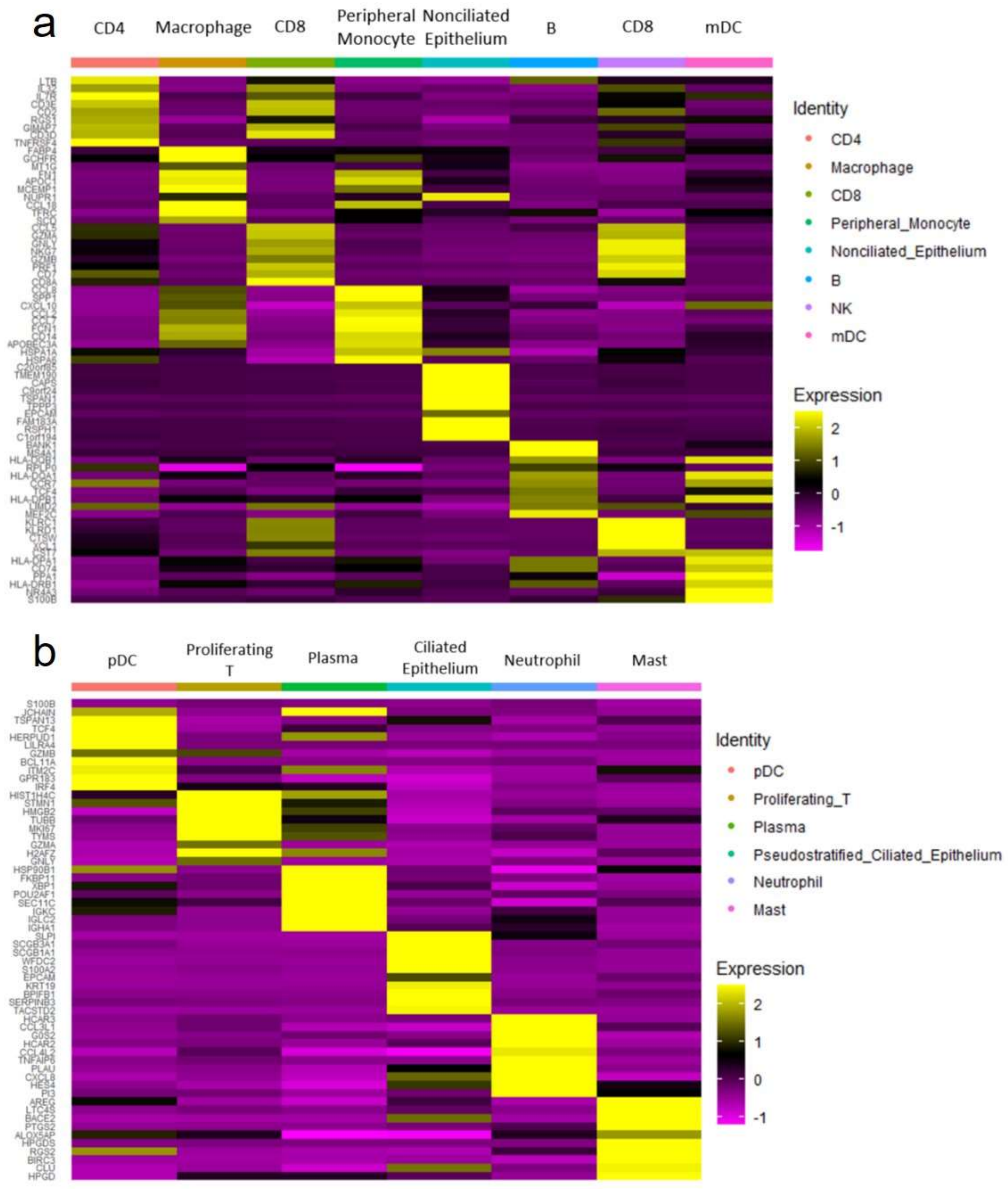
a
CD4
Macrophage
CD8
Peripheral Monocyte
Nonciliated Epithelium
B
CD8
mDC
LTB
IL7R
CD3E
CD2
RGS1
GIMAP7
CD3D
TNFRSF4
FABP4
GCHFR
MT1G
FN1
APOC1
MCEMP1
NUPR1
CCL18
TFRC
CCL5
GZMA
GNLY
NKG7
GZMB
PRF1
CD7
CD8A
CCL8
SPP1
CXCL10
CCL2
CCL7
FCN1
CD14
APOBEC3A
HSPA1A
HSPA6
C20orf85
TMEM190
CAPS
C9orf24
TSPAN1
TPPP3
EPCAM
FAM183A
RSPH1
C1orf194
BANK1
MS4A1
HLA-DQB1
RPLP0
HLA-DQA1
CCR7
TCF4
HLA-DPB1
LIMD2
MEF2C
KLRC1
KLRD1
CTSW
XCL1
CST7
HLA-DPA1
CD74
PPA1
HLA-DRB1
NR4A3
S100B
Identity
CD4
Macrophage
CD8
Peripheral_Monocyte
Nonciliated_Epithelium
B
NK
mDC
Expression
2
1
0
-1
b
pDC
Proliferating T
Plasma
Ciliated Epithelium
Neutrophil
Mast
S100B
JCHAIN
TSPAN13
TCF4
HERPUD1
LILRA4
GZMB
BCL11A
ITM2C
GPR183
IRF4
HIST1H4C
STMN1
HMGB2
TUBB
MKI67
TYMS
GZMA
H2AFZ
GNLY
HSP90B1
FKBP11
XBP1
POU2AF1
SEC11C
IGKC
IGLC2
IGHA1
SLPI
SCGB3A1
SCGB1A1
WFDC2
S100A2
EPCAM
KRT19
BPIFB1
SERPINB3
TACSTD2
HCAR3
CCL3L1
G0S2
HCAR2
CCL4L2
TNFAIP6
PLAU
CXCL8
HES4
PI3
AREG
LTC4S
BACE2
PTGS2
ALOX5AP
HPGDS
RGS2
BIRC3
CLU
HPGD
Identity
pDC
Proliferating_T
Plasma
Pseudostratified_Ciliated_Epithelium
Neutrophil
Mast
Expression
2
1
0
-1

Supplemental Figure 4. Heatmap displaying canonical gene expression of genes used to annotate unsupervised clusters after Tc reintegration. FindAllMarkers function in Seurat was used to identify the top genes specific to each annotated Tc population. The average gene expression for each Tc subset was generated using the DoHeatmap function in Seurat.

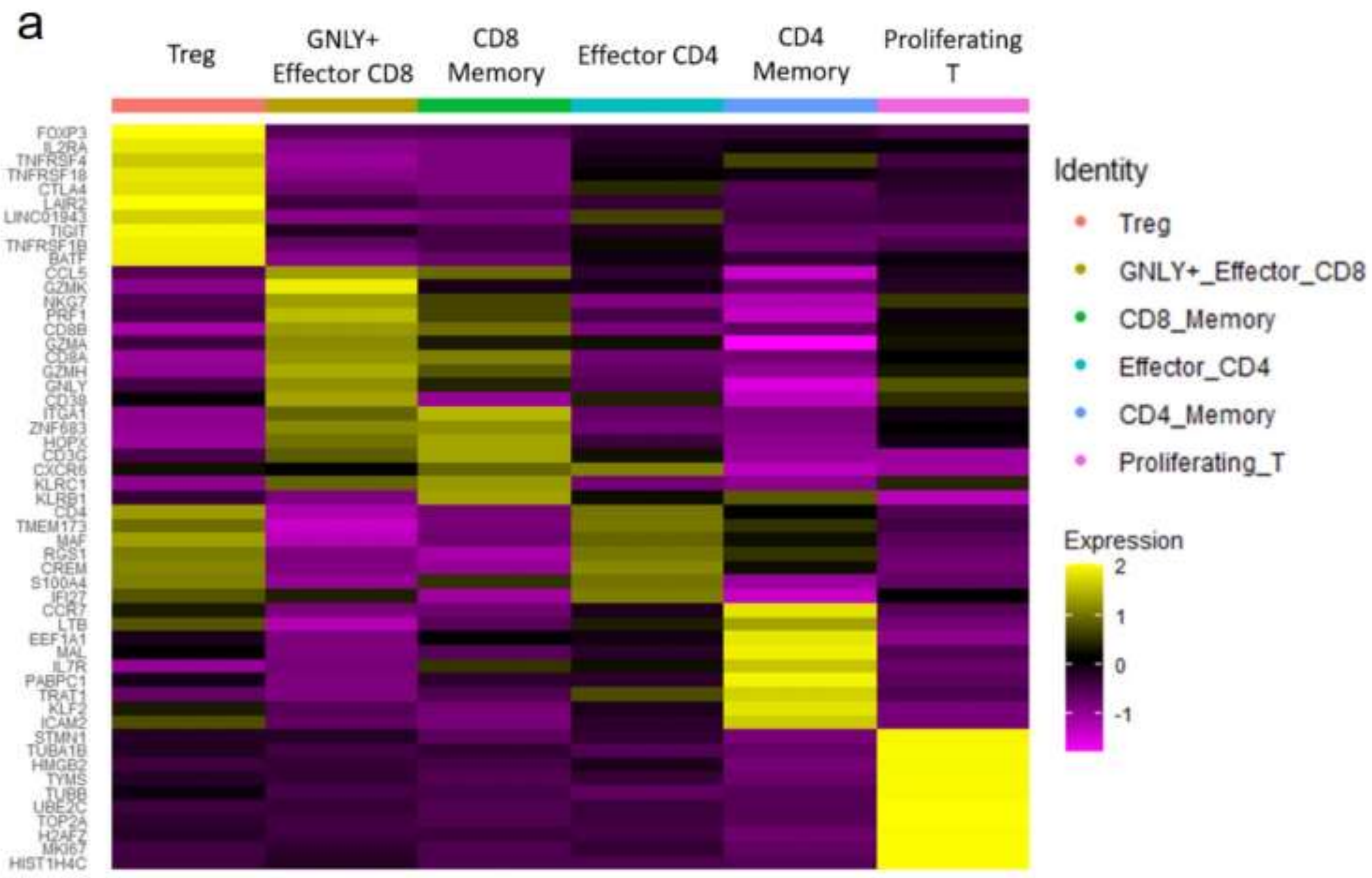

Supplemental Figure 5. Association between prior comorbidities and CD8⁺Tc metabolic dysfunction. Criteria used to define "metabolic syndrome" are in the methodology section. **a**. Summary graphs demonstrating the expression of hif-1a and glut-1 on $CD8^+$Tc in patients with and without metabolic syndrome (n=7 No Met, 10 Met). **b**. Summary graphs demonstrating the expression of hif-1a and lag-3 on $CD8^+$Tc in patients with and without metabolic syndrome (n=11 Norm LDH, 6 Elevated Met). **c.** Matrix demonstrating the Pearson correlation between parameters of elevated LDHA status, elevated CRP status, and serum glucose levels, with expression of ROS, glut-1, vdac, and lag3 in $CD8^+$Tc. Two-tailed student's T-test and Pearson's correlation analysis was done for evaluation of statistical significance. *$p < 0.05$, **$p < 0.01$, and ***$p < 0.001$.

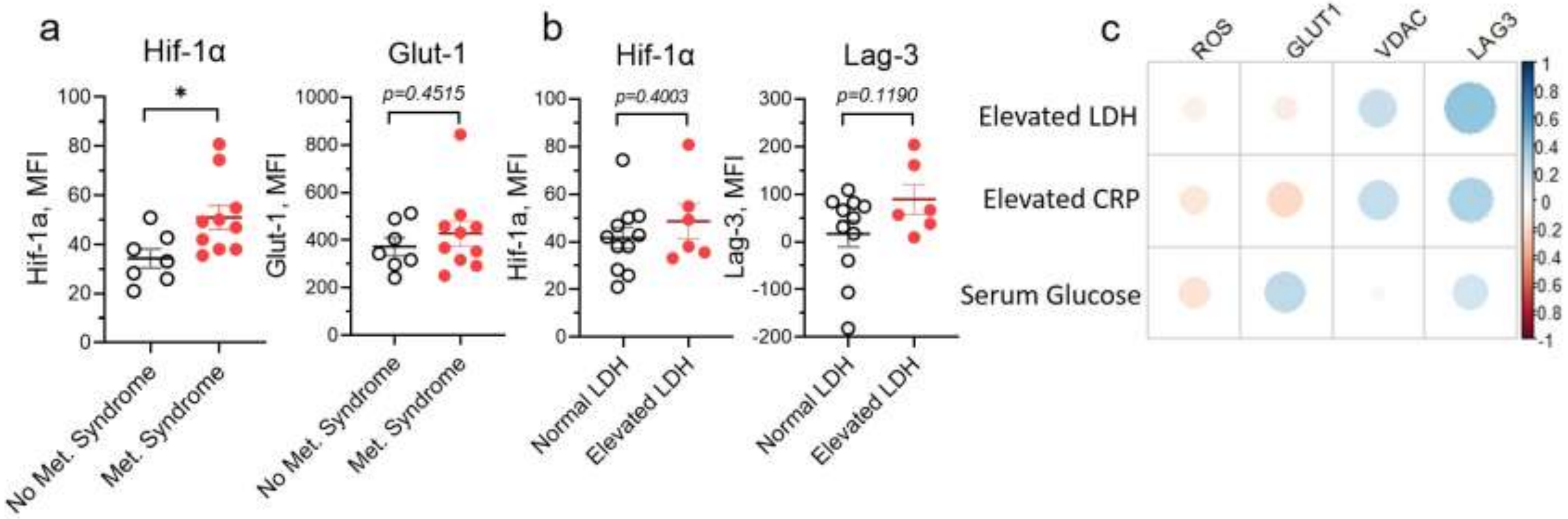

Supplemental Figure 6. Abnormal metabolic phenotype of BALF effector CD8$^+$Tc from severe COVID-19 patients. a. Heatmap displaying expression of key metabolic genes of effector CD8$^+$Tc. (**b-c**) GSEA enrichment and hierarchical clustering plot for glycolysis (**b**) and TCA/respiratory electron transport (**c**) to compare severe COVID-19 vs. healthy control. **d**. Violin plot demonstrating expression of NFE2L2 and PRDX2 across disease states. **e**. UMAP projections unsupervised clustering of patient PBMC scRNAseq data, clusters identified by canonical marker expression to be CD8$^+$Tc were circled manually and labelled. **f**. Heatmap displaying expression of key glycolytic genes differentially expressed in bulk CD8$^+$Tc amongst healthy, mild, and severe COVID-19 patients. **g**. Heatmap displaying expression of key exhaustion genes differentially expressed in bulk CD8$^+$Tc amongst healthy, mild, and severe COVID-19 patients.

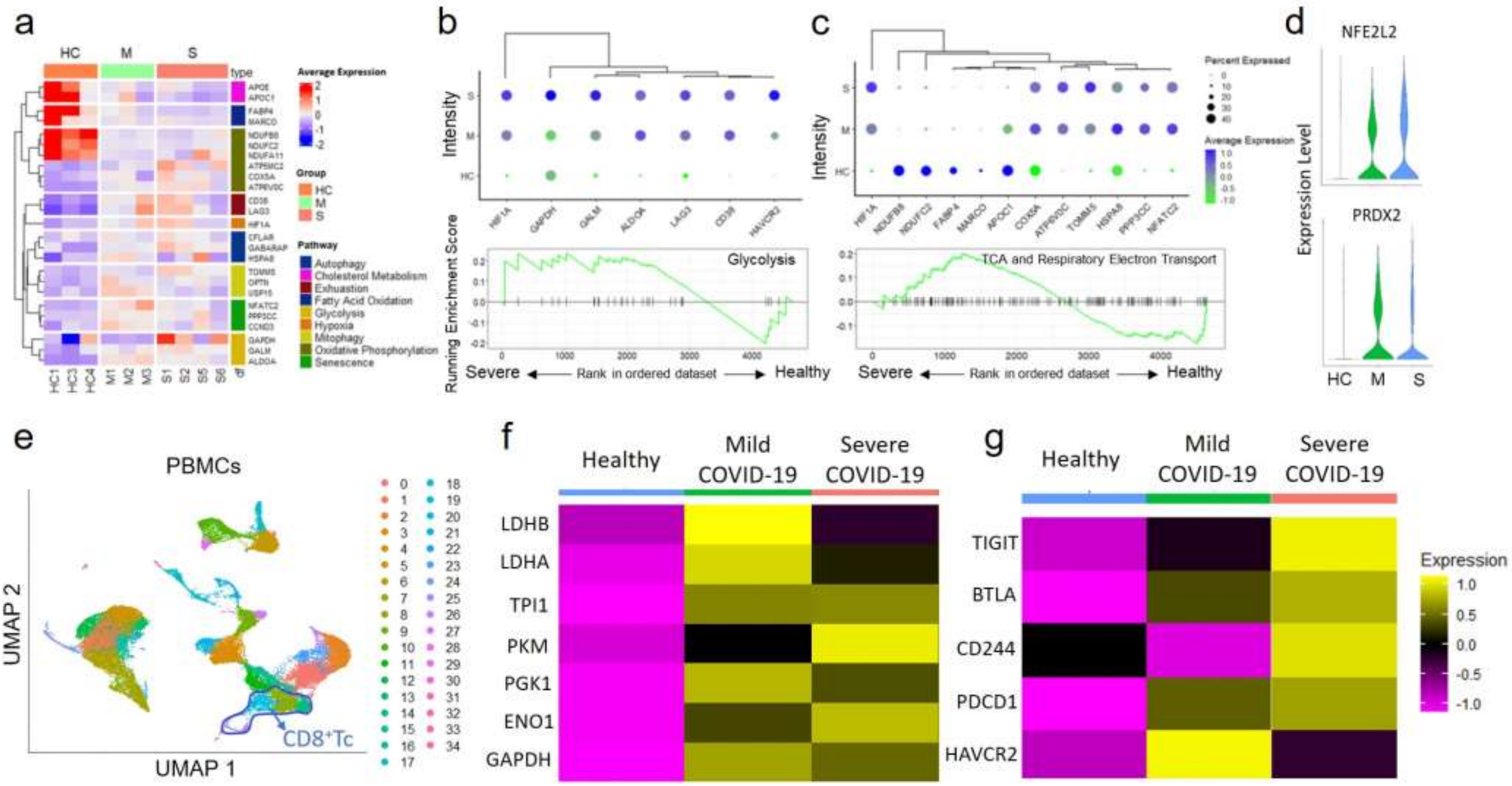

a
HC
M
S
type
APOE
APOC1
FABP4
MARCO
NDUFB8
NDUFC2
NDUFA11
ATP5MC2
COX5A
ATP6V0C
CD38
LAG3
HIF1A
CFLAR
GABARAP
HSPA8
TOMM5
OPTN
USP15
NFATC2
PPP3CC
CCND3
GAPDH
GALM
ALDOA
HC1 HC3 HC4 M1 M2 M3 S1 S2 S5 S6
Average Expression
Group
HC
M
S
Pathway
Autophagy
Cholesterol Metabolism
Exhaustion
Fatty Acid Oxidation
Glycolysis
Hypoxia
Mitophagy
Oxidative Phosphorylation
Senescence
b
Intensity
HIF1A
GAPDH
GALM
ALDOA
LAG3
CD38
HAVCR2
Running Enrichment Score
Glycolysis
Severe
Rank in ordered dataset
Healthy
c
Intensity
HIF1A
NDUFB8
NDUFC2
FABP4
MARCO
APOC1
COX5A
ATP6V0C
TOMM5
HSPA8
PPP3CC
NFATC2
Percent Expressed
Average Expression
TCA and Respiratory Electron Transport
Severe
Rank in ordered dataset
Healthy
d
NFE2L2
PRDX2
Expression Level
HC
M
S
e
PBMCs
UMAP 2
UMAP 1
CD8+Tc
f
Healthy
Mild COVID-19
Severe COVID-19
LDHB
LDHA
TPI1
PKM
PGK1
ENO1
GAPDH
g
Healthy
Mild COVID-19
Severe COVID-19
TIGIT
BTLA
CD244
PDCD1
HAVCR2
Expression

Supplemental Figure 7. Pseudotime and trajectory inference analyses of BALF CD4$^+$Tc during SARS-CoV-2 infection. (**a-b**). UMAP projection (**a**) and dot plot showing pseudotime value (**b**) of 2063 CD4$^+$Tc from reintegrated, healthy, moderate, or severe COVID-19 patients. Each dot represents a single cell.

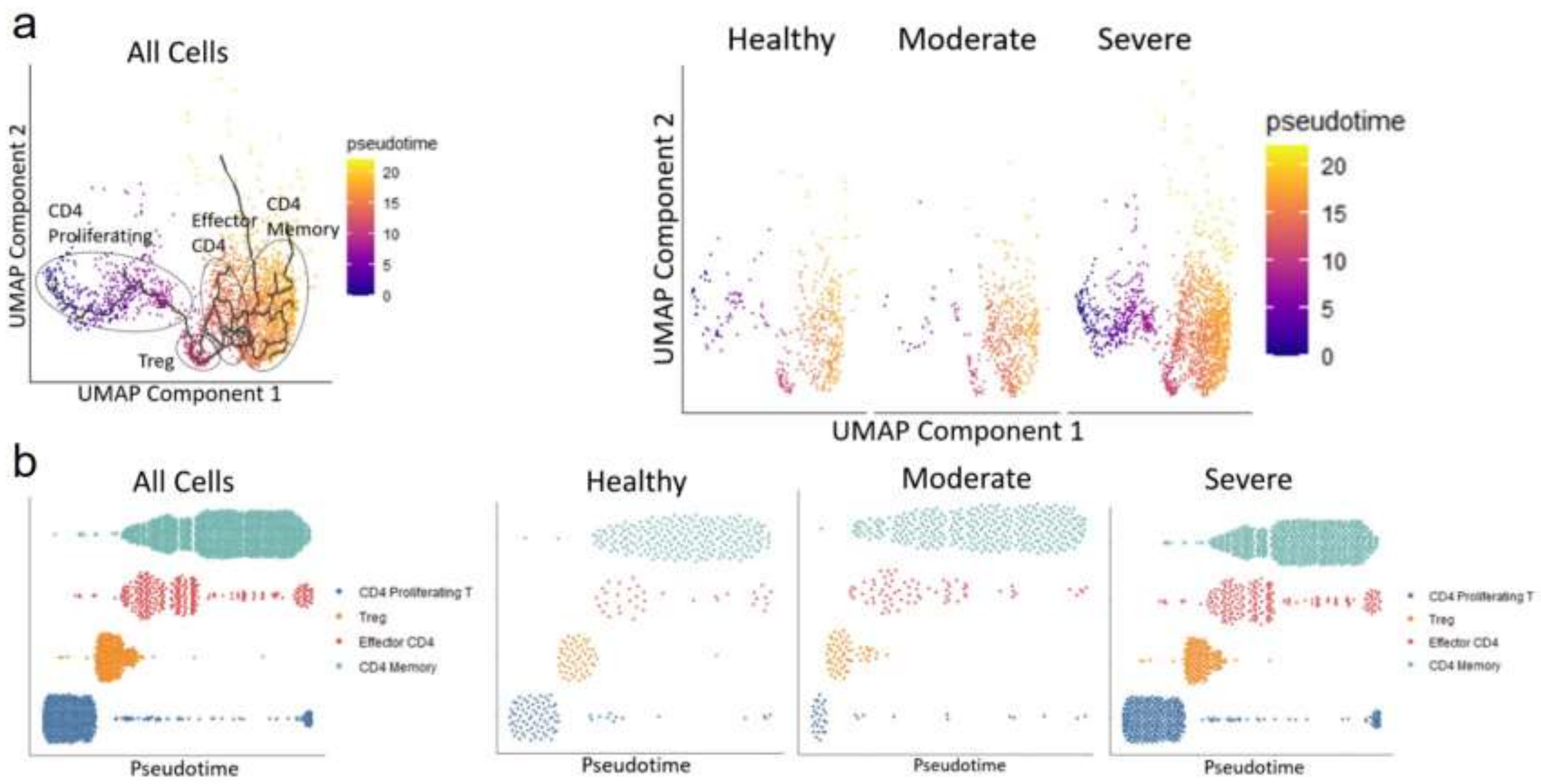

Supplemental Figure 8. BALF NKT are metabolically glycolytic programming in severe COVID-19. A. Heatmap displaying expression of key metabolic genes of NKT. (**b-c**) GSEA enrichment and hierarchical clustering plot for glycolysis (**b**) and TCA/respiratory electron transport (**c**) to compare severe COVID-19 vs. healthy control.

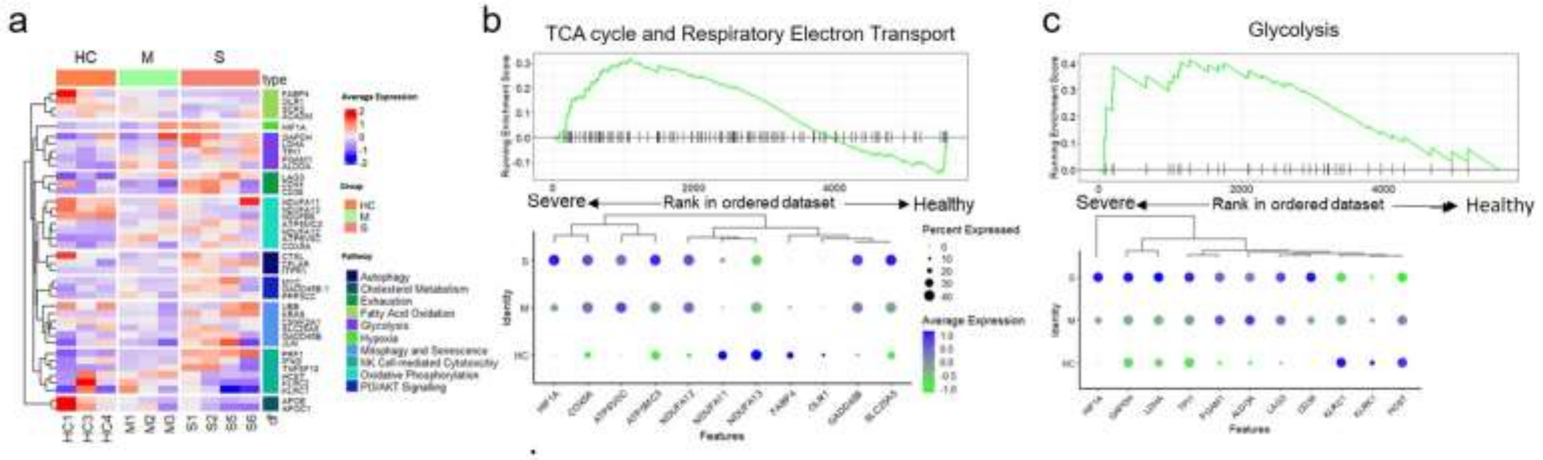

Supplemental Figure 9. Impaired immune surveillance and glycolysis-regulating genes in BALF ECs during SARS-CoV-2 infection. a. Heatmap displaying expression of key genes-regulating immune signaling. **b**. Bar graph showing GSEA analysis of immune signaling pathways, bars are colored by adjusted *p* value. (**c-d**) Linear regression and Pearson correlation analyses for glycolysis and type 1 interferon response (**d**) and NF-kB signaling in ECs (**e**). GSEA enrichment plots for "HLA class 2 antigen presentation", "HLA class 1 antigen presentation", "toll-like receptor cascade", and "interferon α/β response" pathways comparing severe vs. healthy control for pseudostratified ciliated epithelial subset. (**f**) Linear regression and Pearson correlation analyses for glycolysis and HLA Class 2 Signaling. (**g**) Network based display of transcription factor- gene interaction of differentially expressed genes between severe COVID-19 and healthy patients.

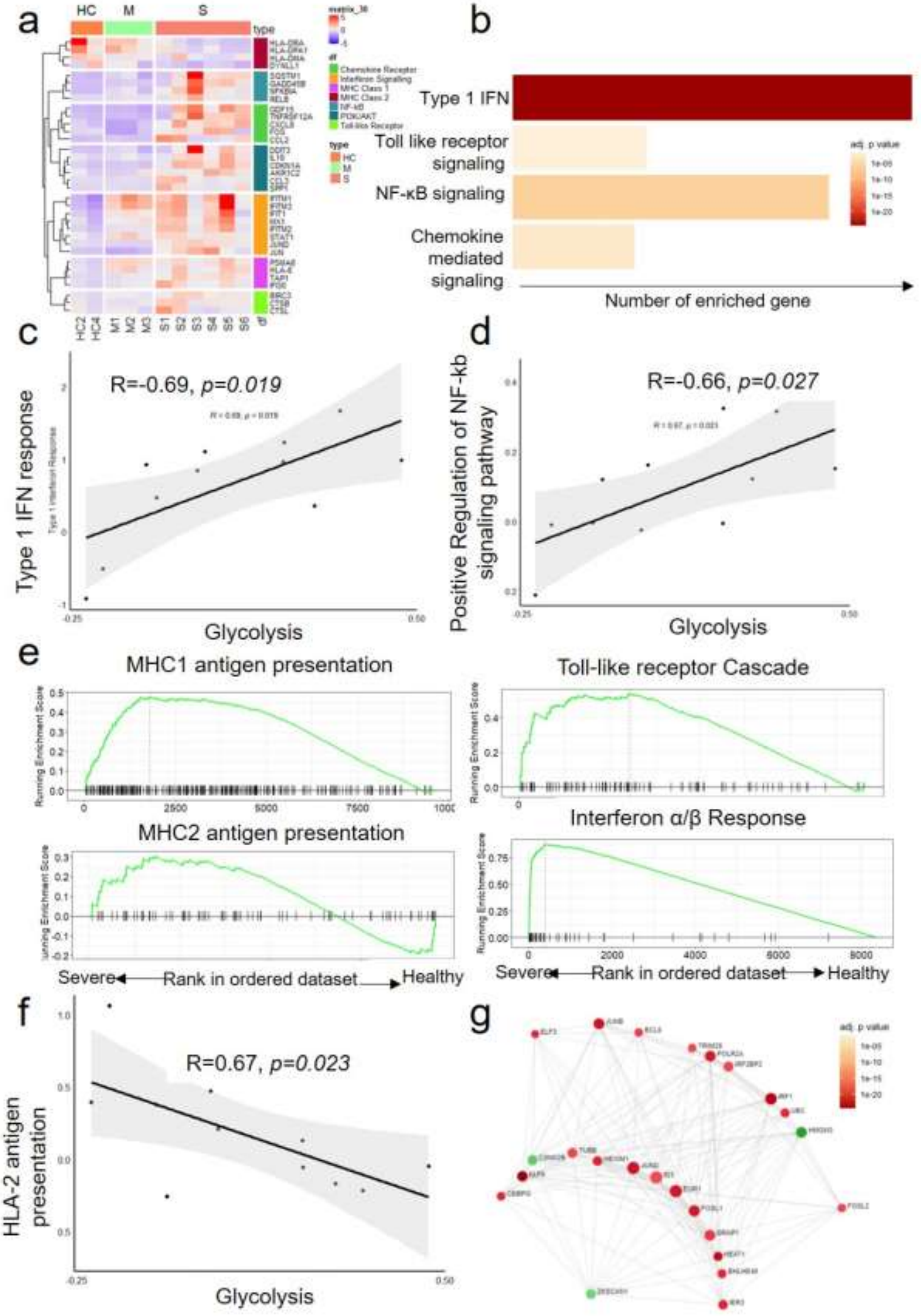
a
HC
M
S
type
b
Type 1 IFN
Toll like receptor signaling
NF-κB signaling
Chemokine mediated signaling
Number of enriched gene
adj. p value
c
R=-0.69, p=0.019
Type 1 IFN response
Glycolysis
d
R=-0.66, p=0.027
Positive Regulation of NF-kb signaling pathway
Glycolysis
e
MHC1 antigen presentation
Toll-like receptor Cascade
MHC2 antigen presentation
Interferon α/β Response
Running Enrichment Score
Severe
Rank in ordered dataset
Healthy
f
R=0.67, p=0.023
HLA-2 antigen presentation
Glycolysis
g
adj. p value

Supplemental Figure 10. Metabolic reprogramming in BALF ECs in severe COVID-19. (**a-b**) UMAP projection of 3531 BALF ECs (**a**) and EC subpopulations (**b**) from healthy, moderate, and severe COVID-19 patients. **b**. UMAP projection of labelled epithelial cell subsets. **c**. Bar graph showing distribution of pseudostratified ciliated and nonciliated ECs. **d**. Heatmap displaying expression of key differentially expressed metabolic genes. **e.** UMAP projection of EC clustered on 42 differentially expressed metabolic genes. **f**. Matrix showing spearman correlation between differentially expressed metabolic genes. (**g-h**) Hierarchical clustering and GSEA enrichment for genes coding for glycolysis (**g**) and for “TCA cycle and respiratory electron transport” (**h**) in ciliated ECs from severe COVID-19 patients and healthy control. (**i-j**) Hierarchical clustering and GSEA enrichment for genes coding for glycolysis (g) and for “TCA cycle and respiratory electron transport” (**h**) in non-ciliated ECs from severe COVID-19 patients and healthy control.

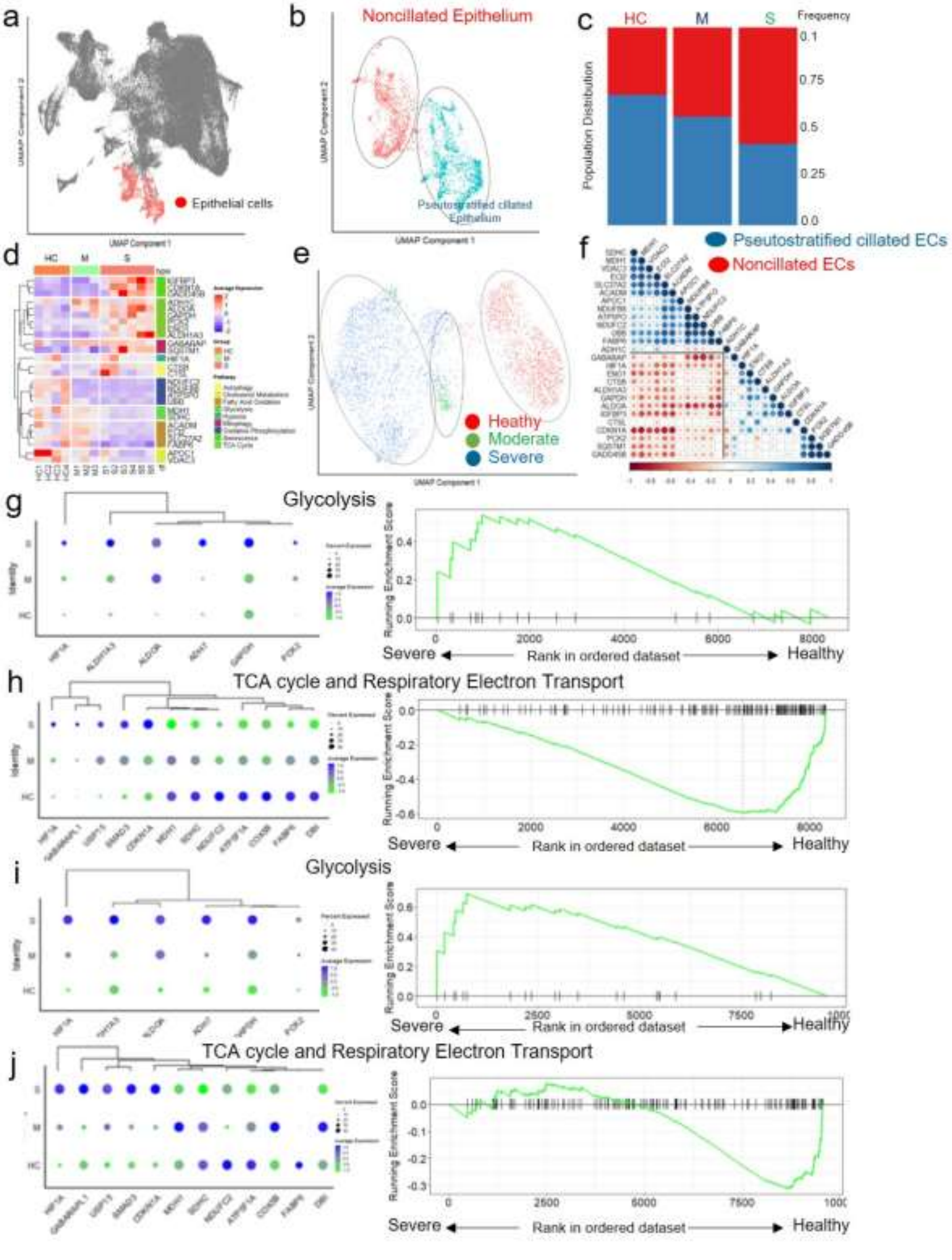
a
Epithelial cells
UMAP Component 1
UMAP Component 2
b
Noncillated Epithelium
Pseutostratified cillated Epithelium
c
HC
M
S
Frequency
Population Distribution
d
e
Heathy
Moderate
Severe
f
Pseutostratified cillated ECs
Noncillated ECs
g
Glycolysis
Running Enrichment Score
Severe
Rank in ordered dataset
Healthy
h
TCA cycle and Respiratory Electron Transport
Running Enrichment Score
Severe
Rank in ordered dataset
Healthy
i
Glycolysis
Running Enrichment Score
Severe
Rank in ordered dataset
Healthy
j
TCA cycle and Respiratory Electron Transport
Running Enrichment Score
Severe
Rank in ordered dataset
Healthy

Supplemental Table 1: Patient Clinical and Demographic Information

Clinical Characteristics	Hospitalized COVID(-) (n = 36)	COVID-19 (n = 52)	*P* value
Average Age (y)	67.74	64.04	0.1792
Male Gender	18/36 (50%)	28/52 (53.85%)	0.8289
Race (White)	22/36 (61.11%)	26/52 (50.00%)	0.3849
Race (Black or African American)	7/36 (19.44%)	13/52 (25.00%)	0.6118
Prior transplantation and/or immunosuppression/dysfunction	5/36 (13.88%)	8/52 (15.38%)	1
Prior type 2 DM/metabolic disorder	20/36 (55.55%)	37/52 (71.15%)	0.1471
Prior hypertension	24/36 (66.66%)	29/52 (55.77%)	0.3776
Prior cardiac arrhythmia	0/36 (0%)	0/52 (0%)	1
Hospitalization	36/36 (100%)	52/52 (100%)	1
Supplemental oxygen or mechanical ventilation	1/36 (2.77%)	7/52 (13.46%)	0.134
Severe respiratory impairment	12/36 (33.33%)	28/52 (53.85%)	0.0814
Presence of dysfunctional lung symptoms	21/36 (58.33%)	40/52 (76.92%)	0.0989
Admission to emergency room	35/36 (97.22%)	50/52 (96.15%)	1
Average time of sample collection (# days after hospitalization)	12.14	14.41	0.403
Laboratory Parameters			
Elevated lactate dehydrogenase	6/36 (16.67%)	17/52 (32.69%)	0.138
Elevated C-reactive protein	14/36 (38.89%)	25/52 (48.08%)	0.5131
Average bedside glucose level (mg/dl)	157.38	140.2128	0.3425

Supplemental Table 2: scRNA SEQ cluster identification markers

Cell type	Panel for metabolic phenotype-based clustering
CTL	APOE, APOC1, FABP4, MARCO, NFUFB8, NDUFC2, NDUFA11, ATP5MC2, COX5A, ATP6V0C, CD38, LAG3, HIF1A, CFLAR, GABARAP, HSPA8, TOMM5, OPTN, USP15, NFATC2, PPP3CC, CCND3, GAPDH, GALM, ALDOA
CD8 Memory	APOE, APOC, OLR1, MARCO, FABP4, GABARAP, CFLAR, HSPA8, CCND3, PPP3CC, NFATC2, DGLUCY, GLUD1, CD38, TIGIT, LAG3, GAPDH, GALM, GPI, ALDOA, HIF1A, TOMM5, USP15, OPTN, NDUFB8, OSP15, OPTN, NDUFB8, ATP6V1E1, NDUFA1, ATP5MC2, COX5A, ATP6V0C
Epithelial	IGFBP3, CDKN1A, GADD45B, ADH1C, ALDOA, GAPDH, PCK2, ENO1, ALDH1A3, GABARAP, SQSTM1, HIF1A, CTSB, CTSL, NDUFC2, NDUFB8, ATp5PO, UBB, MDH1, SDHC, ACADM, ACI2, SLC27A2, FABP6, APOC1, VDAC3
NKT	FABP4, OLR1, SCP2, ACADM, HIF1A, GAPDH, LDHA, TPI1, PGAM1, ALDOA, LAG3, CD44, CD38, NDUFA11, NDUFA13, NDUFB8, ATp5MC2, NDUFA12, ATP6V0C, COX5A, CTSL, CFLAR, ITPR1, UBB, KRAS, CSNK2A1, SLC25A5, GAFF45B, JUN, PRF1, IFNG, TNFSF10, HCST, LRC2, KLRC1, APOE, APOC1

Supplemental Table 3: scRNA SEQ genes used for module scores

	Glycolysis Module Score	FAO Module Score	HLA Class 2 Signaling Module Score	Type 1 Interferon Module Score	Nf-kB Module Score
CD8 Memory	GAPDH, GALM, GPI, ALDOA	OLR, MARCO, FABP4			
Epithelial	ADH1C, ALDOA, GAPDH, PCK2, ENO1, ALDH1A3	ACADM, ECI2, SLC27A2, FABP6	HLA-DRA, HLA-DPA1, HLA-DMA, DYNLL1	IFITM1, IFITM2, IFIT1, MX1, IFITM2	SQSTM1, GADD45B, NFKBIA, RELB

Supplemental Table 4: Surface Markers Used for PBMC Cell Subset Identification

PBMC Cell Population	Surface Markers Used for Identification
Cytotoxic T Lymphocytes	$CD8^+$, $GRZMB^+$
CD8 Central Memory Cells	$CD8^+$, $CD62L^+$, and $CCR7^+$, or $CD8^+$, $CD45RA^-$, $CD62L^+$
CD8 Effector Memory Cells	CD8, $CD62L^-$, $CCR7^-$, or $CD8^+$, $CD45RA^-$, $CD62L^-$
NK Cells	CD56
NKT Cells	CD56, CD8
$CD62L^+$ NK Cells (Memory NK)	CD56, CD62L

Supplemental Table 5: Chemical and Reagent List

Antibodies

Reagent/Resource	Source	Identifier
Anti-Human CD8 APC/Cy7	BioLegend	344722
Anti-Human CD4 PECy7	BioLegend	300512
Anti-Human CD137 PE	BioLegend	309804
Anti-Human LAG-3 PE-Dazzle	BioLegend	369332
Anti-Human CD45RA PerCP	BioLegend	304156
Anti-Human GLUT1 AF700	R&D Systems	FAB1418N
Anti-Human CD62L BV510	BioLegend	304844
Anti-Human CD56 BV650	BioLegend	362532
Anti-Mouse/Human Ki67 PE-Dazzle	BioLegend	151220
Anti-Mouse/Human Granzyme B PECy7	BioLegend	372214
Anti-Mouse/Human LC3	BioLegend	848802
Anti-Human CD4 Pacific Blue	BioLegend	300521
Anti-Human CD98 FITC	BioLegend	315603
Anti-Human IFNy PECy7	BioLegend	502528
Anti-Human TNF-alpha APC	BioLegend	502912
Anti-Mouse/Human CPT1a	Santa Cruz Biotechnology	sc-393070
Anti-Human CD8 FITC	BioLegend	980908
Anti-Human HIF-1-alpha APC	R&D Systems	IC1935A
Anti-Mouse/Human VDAC	Alomone Labs	AVC-001
Anti-Human CCR7 BV510	BioLegend	353232
Anti-Human H2DCFDA	ThermoFisher	D399
Anti-Rabbit Antibody FITC	Southern Biotechnology Associates	4030-02
Anti-Mouse Antibody FITC	Invitrogen	F-2761

Chemicals

Reagent/Resource	Source	Identifier
PBS	Fisher Scientific	BP3994
DMSO	Thermo Scientific	036480.K2
2-DG	Acros Organics	1.12E+08
Oligomycin A	Cayman	11342
Mdivi-1	Cayman	15559
Fetal Bovine Serum, Heat-inactivated	Sigma	F4135-500ML
Sodium Azide	Acros Organics	19038-1000
BSA	GoldBio	A-420-100
FAM-Dc-Puromycin	Jena Bioscience	NU-925-6FM-S
2-NBDG	Invitrogen	N13195
TMRM	ThermoFisher	T668
Mitotracker	Cell Signaling	9082
BODIPY	Invitrogen	C2102
Live/Dead	Invitrogen	L34959
PMA	Sigma	P8139
Ionomycin calcium salt from Streptomyces conglobatus	Sigma	I0634
FOXP3 Fix/Perm Buffer	BioLegend	421401
Puromycin	Cayman	13884
Anti-Puromycin AF647	EMD Millipore	MABE343-AF647
RPMI 1640 Medium	Gibco	11875-093

Peptides

Reagent/Resource	Source	Identifier
SARS-Cov-2 CD8 epitope megapool	Drs. Alessandro Sette & Weiskopf	Megapool A [92]
